# Genome-wide association study and genomic prediction of bolting trait in spinach (*Spinacia oleracea* L.)

**DOI:** 10.3389/fpls.2026.1795277

**Published:** 2026-04-20

**Authors:** Hanan Alkabkabi, Kenani Chiwina, Yuejun Qu, Renjie Du, Renuka Khanal, Qun Luo, Muhamad Muneeb Ullah, Haizheng Xiong, Pablo Rosas-Anderson, Beiquan Mou, Ainong Shi

**Affiliations:** 1 Department of Horticulture, University of Arkansas, Fayetteville, AR, United States; 2 80 Acres Farm Company, Springdale, AR, United States; 3 Sam Farr United States Crop Improvement and Protection Research Center, U.S. Dept. of Agriculture, Agricultural Research Service (USDA-ARS), Salinas, CA, United States

**Keywords:** bolting, floral transition, genome-wide association study (GWAS), genomic prediction (GP), single-nucleotide polymorphism (SNP), spinach, *Spinacia oleracea* L.

## Abstract

Spinach (*Spinacia oleracea* L.) is a major leafy vegetable valued for its nutritional content and commercial importance. The timing of bolting, defined as the transition from vegetative to reproductive growth, is a critical factor determining harvest period and leaf quality. In this study, the objectives were to identify genomic regions associated with bolting variation and assess genomic prediction (GP) accuracy for molecular breeding. Evaluation of bolting in a panel of 295 United States Department of Agriculture (USDA) accessions revealed a bimodal distribution reflecting contrasting bolting phenotypes. Whole-genome resequencing (WGR) yielded 16,563 high-quality SNPs. A multi-model GWAS approach identified seven significant loci distributed across chromosomes 2, 4, and 6. A major-effect locus was identified on chromosome 6 (~13.54 Mb), where the lead SNP, SOVchr6_13545882, exceeded the significance threshold with a peak −log_10_(P) value of 8.66. Consistently identified across multiple robust models, this SNP explained 21.18% of the phenotypic variance (PVE). Within this interval, candidate genes *SOV6g004520* (cysteine-rich receptor-like kinase) and *SOV6g004560* (PPR protein) were prioritized due to their established roles in floral transition pathways. GP analysis further indicated a predominantly additive genetic architecture, with the rrBLUP model achieving a peak predictive accuracy of *r* ≈ 0.39. A prioritized set of only six significant GWAS-derived SNPs (m6) achieved accuracy levels equivalent to the whole-genome panel. The genomic regulation of bolting identified through this analysis establishes a foundation of validated resources for the development of spinach cultivars with optimized reproductive timing.

## 1 Introduction

Spinach (*Spinacia oleracea* L.) is a widely cultivated diploid leafy vegetable in the Amaranthaceae family and a major component of global vegetable production, with annual worldwide output exceeding 26 million tons (Safdar et al., 2022; Niu et al., 2023). The crop is extensively grown across temperate and subtropical regions and is valued for its rapid growth, adaptability, and nutritional richness (Koh et al., 2012; Van Treuren et al., 2012; Xu et al., 2017). Spinach provides essential micronutrients such as iron, calcium, magnesium, and folate, and is enriched with carotenoids, flavonoids, and vitamins A and C, making it an important dietary and economic commodity (Chitwood et al., 2016; Ribera et al., 2020). Beyond its nutritional value, spinach serves as a tractable model for studies of dioecy, photoperiod responsiveness, and abiotic stress tolerance due to its diploid genome, short generation time, and broad natural diversity (Abolghasemi et al., 2021; Hirakawa et al., 2021). Productivity and market quality, however, remain highly sensitive to environmental cues, particularly premature bolting, a rapid transition from vegetative to reproductive growth that shortens the harvest period and reduces consumer acceptability (Abolghasemi et al., 2021; Meng et al., 2022).

Bolting in spinach is primarily induced by long-day photoperiods and elevated temperatures that accelerate stem elongation and floral initiation (Chun et al., 2001). Classical physiological studies demonstrated that manipulating day length and temperature strongly influences bolting onset (Chun et al., 2001), while recent transcriptomic analyses have revealed substantial variation among cultivars in their bolting tendencies under field conditions, reflecting interactions between genotype and environment (Wu et al., 2024). While bolting is important for spinach seed production, agronomic performance is closely tied to bolting behavior, as early bolting reduces leaf quality and marketability, whereas late-bolting cultivars support longer harvest windows and improved texture and flavor (Wu et al., 2024). Ecological patterns further distinguish early bolting in arid or subtropical regions, where rapid flowering provides a reproductive advantage, from late bolting in temperate climates with extended growing seasons (Meng et al., 2022).

Flowering induction in long-day plants involves interconnected photoperiodic, vernalization, and hormonal pathways that converge on conserved transcriptional modules. Central to these processes is the *CONSTANS* (*CO*) and *FLOWERING LOCUS T* (*FT*) regulatory system, in which *CO* integrates circadian and light signals to activate FT, a mobile florigen that promotes *SOC1* and *AP1* expression in the shoot apical meristem (Andrés and Coupland, 2012; Pin et al., 2012; Song et al., 2015). Spinach encodes homologs of these regulators, and genes such as *SoCOL*, *SoFT1*, and *SoFT2* exhibit photoperiod-responsive expression consistent with conservation of *CO-FT* signaling (Meng et al., 2022). Additional pathways involving vernalization and gibberellin signaling influence the expression of *FLC* (Flowering Locus C), *GA20ox*, and *GA3ox*, contributing to variation in bolting behavior across accessions (Wu et al., 2024).

Spinach is mostly dioecious and outcrossing, which contributes to extensive nucleotide polymorphism and rapid linkage disequilibrium decay of approximately 100 kb, characteristics that enable high-resolution genetic mapping (Chitwood et al., 2016). Population-structure analyses consistently identify three major genetic groups known as Asian, European, and American, reflecting domestication history and regional adaptation (Abolghasemi et al., 2021). This broad genetic diversity, together with rapid linkage disequilibrium (LD) decay, makes spinach particularly well suited to genome-wide association studies (GWAS) and genomic prediction (GP) analyses targeting complex agronomic traits such as bolting.

High-quality reference genomes such as Monoe-Viroflay and SP75 have accelerated gene discovery and trait dissection in spinach (Cai et al., 2018; Hirakawa et al., 2021). Transcriptomic analyses have identified differentially expressed *FT*, *CONSTANS-like* (*COL*), MADS-box genes, and several hormone- and stress-responsive regulators during floral transition (Abolghasemi et al., 2021; Wu et al., 2024). Quantitative trait locus studies have mapped genomic regions associated with bolting trait and highlighted *FT*- and *COL*-related candidate genes (Meng et al., 2022). Additional resequencing- based mapping has refined these loci and expanded the catalog of candidate flowering regulators across diverse germplasm sets (Meng et al., 2022).

GWAS utilizes historical recombination to identify loci underlying quantitative traits and has proven powerful in spinach genetic research. Previous GWAS have identified single-nucleotide polymorphisms associated with bolting, leaf morphology, mineral content, and disease resistance (Chitwood et al., 2016; Abolghasemi et al., 2021; Wu et al., 2024). However, relatively few studies have integrated GWAS with GP, an approach that estimates genomic breeding values using genome- wide markers (Heffner et al., 2009; Crossa et al., 2017). Evidence from cereals and legumes demonstrates that combined GWAS and GP frameworks improve prediction accuracy for polygenic traits such as flowering time and yield (Spindel et al., 2016; Crossa et al., 2017; Mora-Poblete et al., 2023). Given the moderate heritability and largely additive genetic control of bolting (Meng et al., 2022), integrating these approaches is particularly suitable for spinach improvement.

The present study had two primary objectives: (1) to perform a GWAS to identify single nucleotide polymorphism (SNP) markers associated with bolting trait in spinach, and (2) to implement GP models to evaluate the ability of these markers to predict bolting performance across diverse accessions. To achieve these goals, we analyzed a panel of 295 United States Department of Agriculture (USDA) spinach accessions genotyped with 16,563 high-quality SNPs generated through whole-genome resequencing (WGR). By integrating GWAS with GP, the findings contribute to a clearer understanding of the genomic architecture underlying bolting trait, which may support molecular breeding programs, guide the development of improved spinach cultivars, and provide a foundation for future work on the genomic regulation of bolting in spinach and other vegetable crops.

## 2 Materials and methods

### 2.1 Plant materials and phenotypic evaluation

A total of 295 spinach (*Spinacia oleracea* L.) accessions were obtained from the USDA Germplasm Resources Information Network (GRIN) maintained at the North Central Regional Plant Introduction Station (NCRPIS), Ames, Iowa, USA. The collection represented germplasm from 29 countries, with the largest contributions from Turkey (95 accessions, 32.2%), the United States (49 accessions, 16.6%), and Afghanistan (20 accessions, 6.8%) (**Supplementary Table 1**).

The phenotypic evaluation was conducted at the USDA-ARS Research Station in Salinas, California, following the experimental protocol established by Mou (2008). To assess bolting under natural ambient conditions, the population was maintained in an outdoor insect-proof cage (screen house) from August to October. During the trial period, the plants were exposed to the characteristic seasonal fluctuations of the Salinas Valley; average high temperatures ranged from 22.8 °C (73°F) to 23.9 °C (75°F), while average low temperatures transitioned from 12.2 °C (54°F) in August to 9.4 °C (49°F) in October. The natural photoperiod decreased from 13.5 to 11.2 hours.

The experiment was conducted as a single-trial (unreplicated) diversity screening. Accessions were grown in plastic pots (10 × 10 × 10 cm) with a 2:1 (v/v) sand-soil mixture. To ensure uniform plant density and vigor, sixteen seeds per accession were initially sown and subsequently thinned to 10 healthy, representative plants per pot.

Bolting trait was evaluated based on stem elongation status at the time of phenotypic assessment and used to classify accessions as early bolting if stem elongation had occurred before 60 days after planting (score = 1), intermediate if elongation was observed between 60 and 70 days (score = 5), or late bolting if elongation had not yet occurred by 70 days after planting (score = 9). Scoring criteria followed the standards of Chitwood et al. (2016) and USDA-GRIN (https://npgsweb.ars-grin.gov/gringlobal/method?id=492382). To minimize environmental noise in this unreplicated trial, the arithmetic mean of the 10 plants per accession was calculated and utilized as the phenotypic value for downstream genomic analyses, including GWAS and genomic prediction, following the established approach of analyzing ordinal and binary phenotypic data using genomic tools such as GAPIT (Wang and Zhang, 2021; Alavilli et al., 2022; Bagwell et al., 2025).

Genomic heritability ( $h_{2}g$) was estimated using the ridge regression best linear unbiased prediction (rrBLUP) model (Endelman, 2011). The calculation was based on the genomic relationship matrix (K matrix) (VanRaden, 2008) derived from 16,563 high-quality SNP markers to determine the proportion of phenotypic variance attributed to additive genetic effects (V_g_) relative to the residual variance (V_e_).

### 2.2 DNA extraction, sequencing, and genotyping

Young leaf tissues from 5–10 plants per accession were pooled for genomic DNA extraction to obtain a representative consensus genotype for each accession. Genomic DNA was then extracted using the modified cetyltrimethylammonium bromide (CTAB) method (Doyle, 1990). DNA was sheared to an average fragment size of 350 bp with a Covaris ultrasonic processor, and sequencing libraries were prepared according to Van Dijk et al. (2014). WGR was performed on the Illumina NovaSeq platform (paired-end mode) at an average depth of ~10× per sample, producing ~10 Gb of clean reads per accession.

Raw reads were initially aligned to the *Spinacia oleracea* SP75 genome, then re-aligned to ensure read specificity. Subsequently, reads were re-aligned to the more contiguous Monoe-Viroflay reference genome (Cai et al., 2018; Hirakawa et al., 2021) to maximize mapping precision and SNP discovery accuracy. Both reference genomes were obtained from SpinachBase (http://www.spinachbase.org/) using BWA v0.7.8 (Li and Durbin, 2009). The resulting BAM files were sorted and duplicated using SAMtools v0.1.19 (Li and Durbin, 2009) and merged per accession with Picard v1.111 (Broad Institute, Cambridge, MA). Variant discovery, including SNPs and small insertions/deletions (InDels), were performed using GATK v3.5 (McKenna et al., 2010).

Across all resequenced spinach accessions, ~0.5 million raw SNPs were detected. For the 295 accessions included in this study, variants were filtered using the following criteria: minor allele frequency (MAF) > 0.05, missing rate < 7%, and heterozygosity < 15%. The remaining missing genotypes were imputed using the default mean imputation method in GAPIT version 3 (GAPIT3) (Wang and Zhang, 2021) implemented in R v4.4.2 (R Core Team, 2021). After filtering, 16,563 high quality SNPs remained, distributed across all six spinach chromosomes. SNP counts per chromosome were: 2,629 (Chr1), 2,029 (Chr2), 3,224 (Chr3), 1,870 (Chr4), 3,284 (Chr5), and 3,527 (Chr6) (**Supplementary Figure 1**). SNP density plots were generated using the CMplot package in R v4.4.2 (R Core Team, 2021). All variant data have been deposited in the Figshare repository (https://doi.org/10.6084/m9.figshare.30983827).

### 2.3 Population structure and genetic diversity

Population structure was analyzed using GAPIT version 3 (GAPIT3) (Wang and Zhang, 2021) implemented in R v4.4.2 (R Core Team, 2021), based on a dataset of 16,563 SNPs across 295 spinach accessions. Population structure was assessed using principal component analysis (PCA) and neighbor-joining (NJ) phylogenetic analysis. PCA was performed to summarize genome-wide genetic variation, with the number of principal components evaluated ranging from two to ten based on previous studies. NJ phylogenetic trees were constructed using pairwise genetic distances, and subgroup numbers ranging from two to ten were examined to assess clustering consistency.

### 2.4 GWAS analysis

GWAS was performed on bolting trait phenotypes from 295 accessions using 16,563 SNPs. The analysis was conducted in GAPIT3 (Wang and Zhang, 2021) using a Linear Mixed Model (LMM) framework represented by the equation:


Y=Xβ+Zu+e

In this model, *Y* is the vector of mean phenotypic scores (1, 5, and 9) derived from 10 uniform plants per accession. To control for confounding factors, the ‘Q+K’ approach (Yu et al., 2006) was implemented as follows: the population structure (Q matrix), represented by the first three principal components along with the intercept (overall mean), was included as a fixed effect (*β*) via the design matrix X; the kinship matrix (K matrix) was incorporated as a random effect (*u*) via the design matrix *Z*; an *e* represents the vector of residual errors.

To ensure the robustness of the results and the consistency of the identified significant SNPs across different statistical assumptions, five complementary models were utilized for comparative validation Lipka et al., 2012). These models included the Bayesian-information and Linkage-disequilibrium Iteratively Nested Keyway (BLINK) model (Huang et al., 2019), the Fixed and Random Model Circulating Probability Unification (FarmCPU) model (Liu et al., 2016), the multiple-loci mixed linear model (MLMM) (Segura et al., 2012), the generalized linear model (GLM) (Nelder and Wedderburn, 1972), and the mixed linear model (MLM) (Zhang et al., 2010).

SNPs were declared significant when their −log_10_(*P)* values (where *P* represents the SNP–trait association *P*-value) met or exceeded the Bonferroni-corrected genome-wide significance threshold (Bland and Altman, 1995). A Bonferroni correction (α = 0.05) was applied to account for multiple testing by dividing α by the total number of SNP markers (16,563). Accordingly, the significance threshold was calculated as −log_10_ (0.05/16,563), resulting in a final threshold of −log_10_(*P*) > 5.52.

The proportion of phenotypic variance explained (PVE) for each significant SNP was estimated using the GAPIT3 package outputs. Allelic effects of significant SNPs were evaluated using Fisher’s exact test (Kaplan and Weir, 1992), which is appropriate for a binary ordinal trait (early = 1, late = 9), to compare early/late category frequencies between homozygous allelic classes.

### 2.5 LD analysis and candidate gene identification

LD between SNP loci was quantified as the squared allele-frequency correlation (*r*^2^) using TASSEL version 5 (Bradbury et al., 2007). LD decay was evaluated using 16,563 SNP markers across 295 spinach accessions using two LD-based strategies. First, LD decay was assessed at the chromosomal level by examining the relationship between pairwise *r*^2^ values and physical distance (bp) for each chromosome. Second, local LD patterns surrounding significant GWAS SNPs were evaluated by plotting pairwise *r*^2^ values against physical distance within SNP-specific genomic regions (extending between ± 100 to ± 320 kb from each lead SNP, depending on the chromosome). The LD decay distance was defined as the chromosomal distance at which *r*^2^ declined to half of its maximum value, following established approaches (Kim et al., 2007; Lam et al., 2010). For genomic regions where local LD decay was not estimable, a fixed window of ± 50 kb was applied to define the candidate gene interval, based on the average chromosome-level LD decay distance (Alatawi et al., 2025). Additionally, LD heatmaps and haplotype block structures were visualized with Haploview v4.2 (Barrett et al., 2005).

Candidate genes were identified within genomic regions defined by linkage disequilibrium (LD) surrounding significant GWAS SNPs using two LD-based approaches. When local LD decay could be reliably estimated for an individual SNP, SNP-specific LD decay distances derived from the relationship between pairwise *r²* values and physical distance were used to delineate candidate regions. When local LD decay could not be estimated due to insufficient LD information in the surrounding region, chromosome-level LD decay estimates were applied to define the candidate gene window. Gene coordinates and annotations were obtained from the Monoe-Viroflay spinach reference genome (Hirakawa et al., 2021) using the General Feature Format version 3 (GFF3) annotation file accessed through SpinachBase (http://www.spinachbase.org/).

### 2.6 GP analysis

GP for bolting trait was conducted using three marker-set strategies: (i) random SNP subsets, (ii) GWAS-derived SNP sets obtained from the entire population, and (iii) GWAS-assisted marker sets identified within training populations (80%). Seven statistical models were evaluated, including Bayes A (BA) and Bayes B (BB) (Meuwissen et al., 2001), Bayesian LASSO (BL) and Bayesian Ridge Regression (BRR) (De los Campos et al., 2013), ridge-regression BLUP (rrBLUP) (Endelman, 2011). These parametric models were implemented within a Linear Mixed Model (LMM) framework:

Y=Xβ+Zu+e where *Y* is the vector of phenotypic observations, *X* and *Z* are design matrices for fixed and random effects, respectively, *β* represents the fixed effect (overall mean), *u* is the vector of random additive genetic effects, and *e* is the vector of residual errors. For the rrBLUP model, the genomic relationship matrix (G matrix) was constructed using the Van Raden (2008) method. Conversely, Support Vector Machine (SVM) (Cortes and Vapnik, 1995), and Random Forest (RF) (Breiman, 2001) were employed as non-parametric approaches to capture potential non-linear interactions without an explicit assumption of a genomic relationship matrix. All analyses were implemented in R v4.4.2 (R Core Team, 2021). Prediction accuracy was estimated as the Pearson correlation (*r*-value) between observed and predicted phenotypes, and visualization was performed using ggplot2 (Wilkinson, 2011).

#### 2.6.1 GP using different random SNP subsets

Ten random SNP marker subsets of increasing size (6, 50, 100, 200, 500, 1,000, 2,000, 5,000, 10,000, and all 16,563 SNPs), hereafter denoted as r6, r50, r100, r200, r500, r1000, r5000, r10000, and all_16563 SNPs, were randomly sampled from the full dataset using a simple random sampling approach, as previously applied in genomic prediction marker density studies (Ma et al., 2016; Zhang et al., 2017; Li et al., 2024). Each subset was analyzed using the seven genomic prediction models with five-fold cross-validation (training: validation = 4:1). To ensure representative genome-wide coverage and minimize sampling bias, each model–subset combination was replicated 100 times. Prediction accuracy was quantified as the mean *r*-value between observed and predicted phenotypes, with standard errors (SE) calculated across replicates.

#### 2.6.2 GP using GWAS-derived SNPs (whole panel)

Significant SNPs exceeding the significance threshold (−log_10_(*P)* > 5.52) were identified from GWAS analyses conducted on the entire panel of 295 spinach accessions. Based on these results, three GWAS- derived marker sets were constructed, containing two, four, and six SNPs, and hereafter referred to as m2, m4, and m6, respectively. These marker sets were subsequently used as inputs for GP analyses to assess the feasibility of using a cost-effective, low-density marker panel for breeding applications. Following the LMM framework described previously, the effects of these targeted loci were estimated as random variables to evaluate their specific predictive capacity. Prediction accuracy for each marker set was evaluated using the seven GP models described above.

#### 2.6.3 GP using GWAS-derived SNPs from 80% of the whole panel

The full dataset (295 accessions) was randomly divided into a training population (TP, 80%; 236 accessions) and a validation population (VP, 20%; 59 accessions). This partitioning was repeated five times (R1-R5) to create independent TP-VP pairs. GWAS was conducted within each TP using four models (GLM, MLM, FarmCPU, and BLINK) in GAPIT3, applying a moderate significance threshold (-log_10_(*P*) > 3.0) to capture a broader set of trait-associated loci and enhance predictive performance (Spindel et al., 2016; Meng et al., 2022).

To evaluate the robustness and transferability of the GWAS-assisted markers, three prediction schemes were implemented:

1. Across_prediction: SNPs identified in each TP were used to predict bolting trait in the corresponding VP; results were averaged across R1-R5. This scheme specifically evaluates the markers’ predictive power in independent, unseen individuals to ensure they capture stable genetic signals.
2. Cross_prediction: SNPs from all five TPs were combined to predict phenotypes across the entire population (TP + VP) to assess the global stability of the identified loci.
3. Cross_self.prediction: SNPs identified in each TP were used to predict phenotypes within the same TP, serving as a baseline for internal consistency.

Prediction accuracy was estimated from mean *r*-values ± SEs across replicates.

#### 2.6.4 GP using GAGBLUP in GAPIT3 with GWAS-derived SNP markers

Following the genomic prediction strategies described above, GP was further conducted using GWAS-derived significant SNPs identified specifically from the BLINK model (Huang et al., 2019). The BLINK model accounts for linkage disequilibrium (LD) through a multi-locus approach, which minimizes false-positive associations and ensures the selection of high-confidence markers (Huang et al., 2019). SNPs exceeding the predefined significance threshold (−log_10_(*P)* > 5.52) were selected and subsequently used as marker inputs for genomic prediction using the GAGBLUP (Genetic Algorithm-augmented GBLUP) model (Xu et al., 2024) (previously referred to as maBLUP) (Ma et al., 2025) implemented in GAPIT version 3. This framework integrates significant GWAS markers to prioritize high-effect loci alongside the polygenic background, enhancing prediction accuracy for traits influenced by major genetic components such as bolting (Xu et al., 2024; Ma et al., 2025).

GP performance was evaluated under the same three prediction schemes using the previously defined population partitions: (i) across-population prediction, in which SNPs identified from the TP (80%; 236 accessions) were used to predict genomic estimated breeding values (GEBVs) (Selga et al., 2022) in the VP (20%; 59 accessions); (ii) cross-population prediction, where GWAS-derived SNPs were used to predict GEBVs across the entire panel (TP + VP; 295 accessions); and (iii) cross-self prediction, in which SNPs identified within the TP were used to predict GEBVs for the same TP.

Prediction accuracy was quantified using (*r*-value) between observed phenotypes and predicted GEBVs.

## 3 Results

### 3.1 Phenotypic analysis of bolting trait

**Figure 1:**
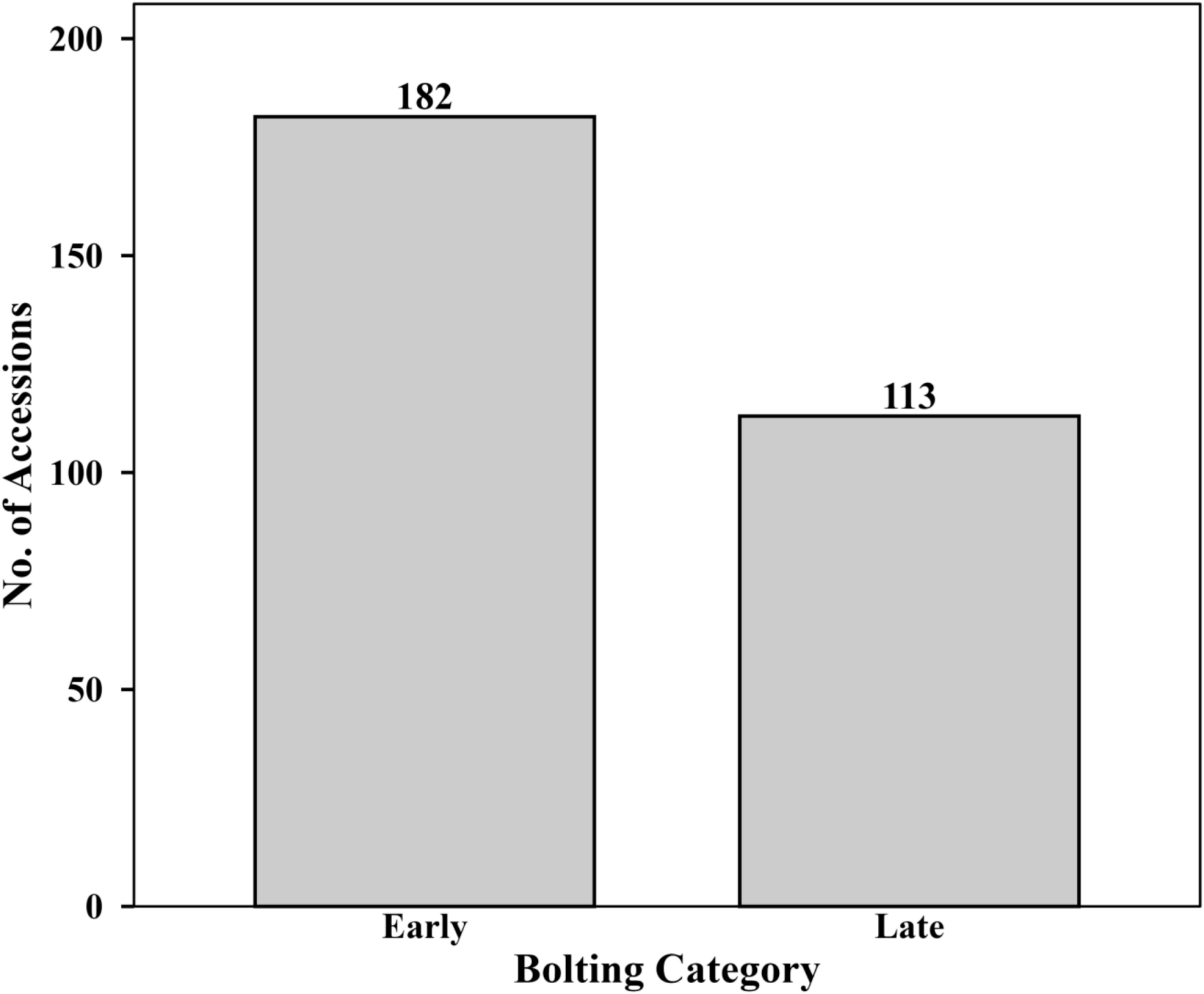
Distribution of bolting categories among 295 spinach accessions. Categories were defined based on days after planting until stem elongation and classified as early (<60 days), intermediate (60–70 days), or late (>70 days).

Bolting trait among the 295 spinach (*Spinacia oleracea* L.) accessions (**Supplementary Table 1**) showed a bimodal distribution, separating the population into early- and late-bolting groups (**Figure 1**). A total of 182 accessions (61.5%) were classified as early bolting (< 60 days after planting), whereas 113 accessions (38.3%) were classified as late bolting (> 70 days). No accessions were recorded within the intermediate range (60–70 days). Early bolting accessions originated mainly from Afghanistan and Syria, whereas late-bolting accessions were primarily from Turkey, North Macedonia, and the United States.

The bolting trait exhibited a genomic heritability of 0.33 (33.13%), with phenotypic variation partitioned into a genetic variance (V_g_) of 5.20 and a residual variance (V_e_) of 10.50, reflecting the significant impact of environmental factors on the phenotypic expression of bolting in this diversity panel.

### 3.2 Population structure and genetic diversity analysis

**Figure 2:**
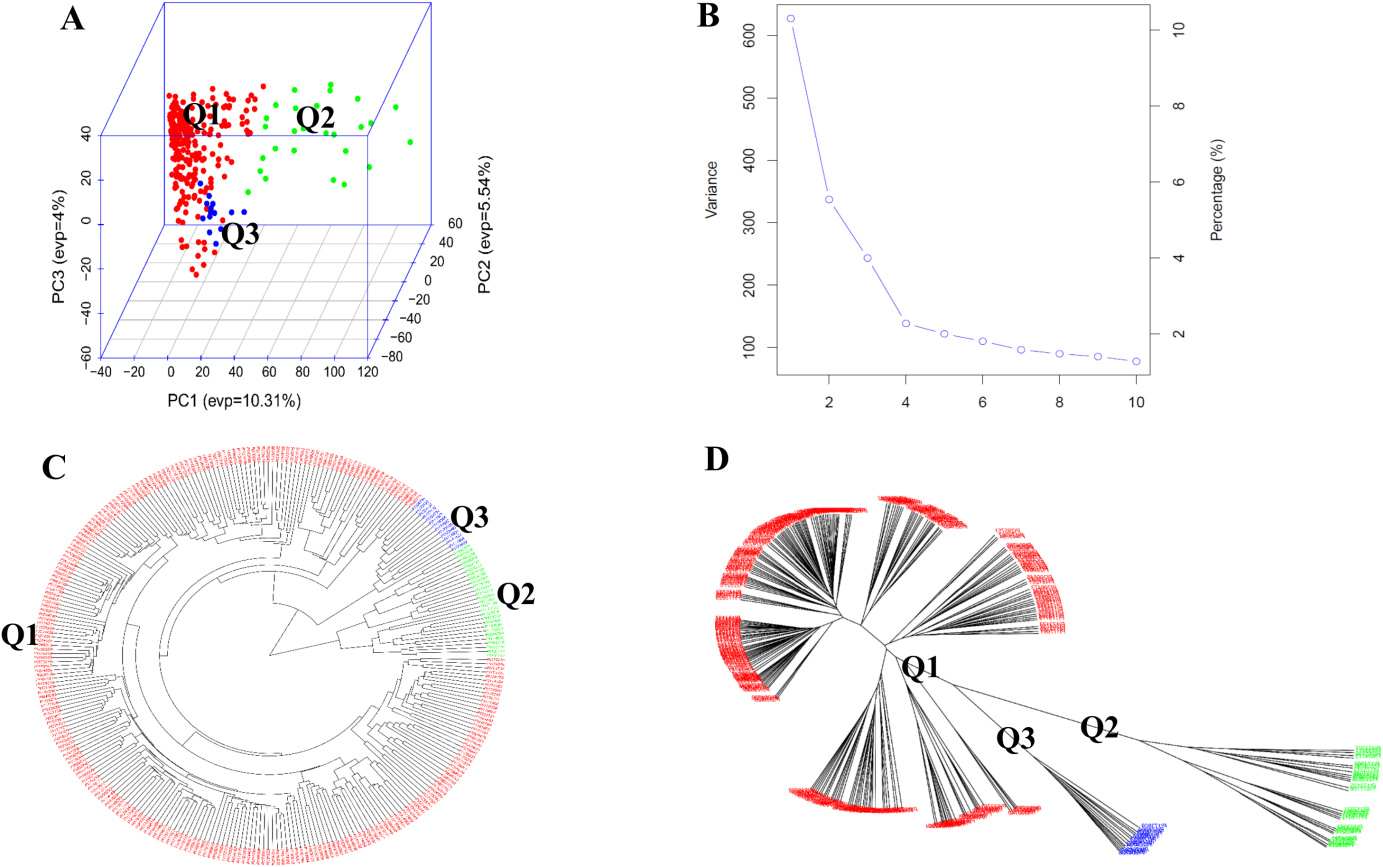
Population structure and genetic diversity of 295 spinach accessions evaluated for bolting trait. **(A)** Three-dimensional principal component analysis (PCA) illustrating genetic clustering of accessions into three sub-populations (Q1 - Q3). **(B)** Eigenvalue plot derived from PCA generated in GAPIT3. **(C, D)** Phylogenetic trees constructed using the neighbor-joining (NJ) method in GAPIT3, shown in fan **(C)** and unrooted **(D)** formats. Each cluster represents a distinct sub-population: Q1 (red), Q2 (green), and Q3 (blue).

PCA based on 16,563 genome-wide SNPs revealed clear genetic stratification among the 295 spinach accessions, separating them into three distinct clusters designated Q1, Q2, and Q3 (**Figure 2A**). The PCA scree plot displayed a pronounced inflection point at the third principal component, indicating that the first three components captured the major structure of genome-wide genetic variation (**Figure 2B**). Together, PC1, PC2, and PC3 explained 10.31%, 5.54%, and 4.04% of the total variance, respectively, accounting for 19.9% of the overall genetic variation.

NJ phylogenetic analysis based on pairwise genetic distances produced clustering patterns consistent with the PCA results, with accessions from Q1 (red), Q2 (green), and Q3 (blue) forming well-defined and distinct clades in both fan-shaped and unrooted tree representations (**Figures 2C, D**).

Geographic origin showed partial correspondence with the inferred genetic structure. Accessions from Central and Western Asia, including Turkey, Afghanistan, and Iran, were predominantly grouped within Q1, whereas accessions from Europe and North America clustered mainly in Q3. Several accessions originating from South and East Asia, particularly from India and China, occupied intermediate positions between Q1 and Q2, suggesting admixed genetic backgrounds. This pattern was consistently observed across PCA and NJ analyses.

### 3.3 GWAS analysis

GWAS analysis was performed using five statistical models (GLM, MLM, MLMM, FarmCPU, and BLINK) implemented in GAPIT3, identifying seven SNPs significantly associated with bolting trait at the genome-wide significance threshold (−log_10_(*P)* > 5.52). These loci were distributed across chromosomes 2, 4, and 6, with a strong enrichment on chromosome 6.

Five of the seven significant SNPs were tightly clustered within a narrow genomic interval spanning 135.45–135.46 Mb on chromosome 6, indicating a major locus influencing bolting trait. Among these, SOVchr6_13545882 exceeded the significance threshold with a maximum −log_10_(*P)* value of 8.66 in the BLINK model and explaining 21.18% of the phenotypic variance (PVE). Two neighboring SNPs, SOVchr6_13545887 (−log_10_(*P)* = 7.97; PVE = 16.23%) and SOVchr6_13545609 (−log_10_(*P)* = 6.00; PVE = 2.51%), were also detected within the same chromosomal region, further supporting the presence of a major-effect locus on chromosome 6.

In addition, SOVchr2_3254529 on chromosome 2 exceeded the significance threshold with a −log_10_(*P)* of 5.83 and accounted for 10.93% PVE, while a single locus on chromosome 4 (SOVchr4_30429857) surpassed the threshold exclusively in the FarmCPU model (−log_10_(*P)* = 5.70), suggesting a model-specific association signal (**Table 1**).

**Table 1:** Seven significant SNPs associated with the bolting trait in spinach, identified using five genome-wide association models in GAPIT3, showing minor allele frequency (MAF), Fisher-test values, and phenotypic variance explained (PVE%).

SNP	Chr	Pos	MAF (%)	GAPIT3						Early allele	Late allele	PVE (%)	Model [-log_10_(*P*-value)] >5.52
				[-log_10_(*P*-value)]									
				BLINK	FarmCPU	MLMM	MLM	GLM	Fisher’s test [-log_10_(*P*-value)]				
SOvchr2_3254529	2	3254529	10.34	5.83	0.81	2.58	1.85	3.39	1.64	C	T	10.93 (BLINK)	BLINK
SOvchr4_30429857	4	30429857	15.08	1.00	5.70	1.10	1.16	0.32	1.11	G	A	–	FarmCPU
SOvchr6_13545571	6	13545571	16.44	0.70	0.61	0.40	4.33	5.92	6.41	T	A	5.05 (GLM)	GLM
SOvchr6_13545584	6	13545584	14.58	0.49	0.43	0.24	4.06	5.75	7.13	T	C	7.81 (GLM)	GLM
SOvchr6_13545609	6	13545609	11.69	0.48	0.62	0.34	4.20	6.00	6.46	T	C	2.51 (GLM)	GLM
SOvchr6_13545882	6	13545882	14.41	8.66	5.66	5.77	5.25	6.86	6.27	C	T	21.18 (BLINK, FarmCPU)	BLINK, FarmCPU, GLM, MLMM
SOvchr6_13545887	6	13545887	14.58	0.03	7.97	0.05	4.79	6.57	5.42	G	A	15.18 (FarmCP); 16.23 (GLM)	FarmCPU, GLM

**Figure 3:**
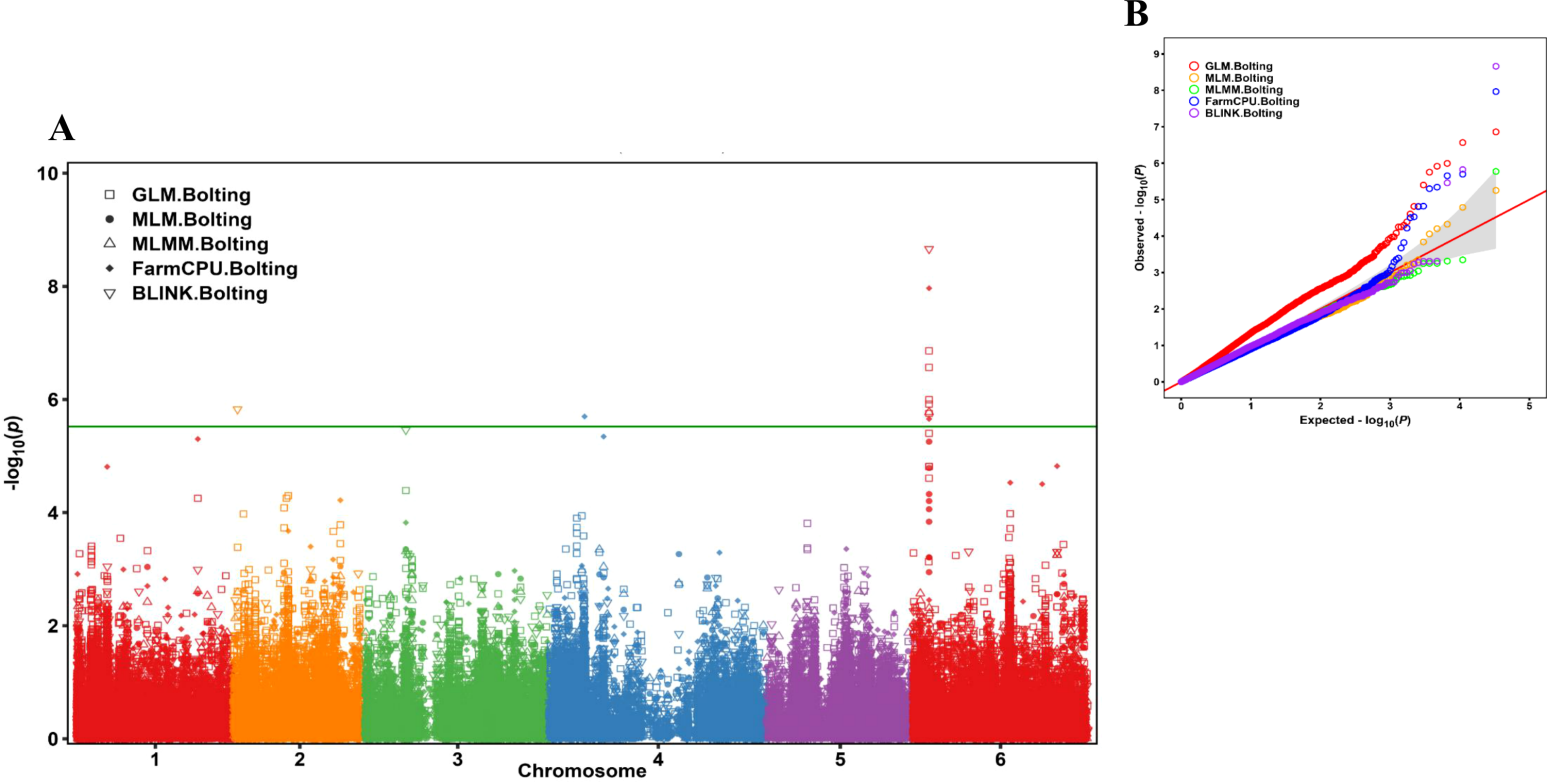
GWAS of the bolting trait in 295 spinach accessions was performed using 16,563 high-quality SNP markers analyzed with five statistical models (GLM, MLM, MLMM, FarmCPU, and BLINK) conducted in GAPIT3. **(A)** The Manhattan plot shows the [-log_10_(*P*-value)] for each SNP across six chromosomes, where each dot represents one SNP marker. SNPs above the green Bonferroni-corrected threshold line (−log_10_ (*P*) > 5.52) are considered significantly associated with the bolting trait. **(B)** The QQ plot displays the expected versus observed −log_10_)*P*(distribution, confirming the reliability of the GWAS models and effective correction for population structure.

Manhattan plots revealed a prominent association peak on chromosome 6, where multiple adjacent SNPs exceeded the significance threshold, consistent across several GWAS models (**Figure 3A****;** **Supplementary Figure 2A**). Quantile–quantile (QQ) plots showed close agreement between observed and expected −log_10_(*P)* distributions, indicating adequate control of population structure and relatedness across all models (**Figure 3B****;** **Supplementary Figure 2B**).

Allelic effect analyses further demonstrated significant differences in bolting trait between early- and late-bolting allelic groups at all seven loci, as confirmed by t-tests (**Table 1**). These findings demonstrate that a major genomic region on chromosome 6 contributes substantially to variation in bolting behavior in spinach and was selected for subsequent linkage-disequilibrium and candidate- gene analyses.

### 3.4 Candidate gene identification

A total of fifteen genes were identified within LD regions associated with seven SNPs significantly linked to bolting trait across chromosomes 2, 4, and 6 (**Supplementary Table 2**). These genes were detected by examining LD-defined genomic intervals surrounding each associated SNP.

Genome-wide LD decay varied across chromosomes (**Supplementary Figure 3A**), and SNP-centered LD analyses showed marker-specific decay distances ranging from approximately 30 kb to 100 kb (**Supplementary Figure 3B**). For the significant SNP on chromosome 4, local LD decay could not be reliably estimated; therefore, annotated genes within a ±50 kb window were examined, consistent with the chromosome-level LD decay distance.

Based on genomic proximity to the associated SNPs and functional annotation, five genes were prioritized as positional candidate genes for bolting regulation (**Table 2**). These candidates were confined to chromosomes 4 and 6, which harbored the strongest and most consistent GWAS signals. On chromosome 4, the associated SNP SOVchr4_30429857 was located upstream of *SOV4g014340*, which encodes a glycine-rich protein and represented the closest annotated gene within the defined interval.

**Table 2:** List of five candidate genes associated with the bolting trait in spinach identified within LD decay regions corresponding to six of the seven significant SNP markers listed in **Table 1**.

Gene	Chr	Gene start_pos (bp)	Gene end_pos (bp)	Gene size (bp)	Gene annotation	SNP	SNP_Pos (bp)	Chr	Distance from gene start (bp)	Distance from gene end (bp)	Comment
*SOV4g014340*	4	30455285	30456105	3259	Glycine-rich protein	SOVchr4_30429857	30429857	4	-25428	-26248	25 kb
*SOV6g004520*	6	13258268	13259050	783	Cysteine rich receptor like kinase	SOVchr6_13545571	13545571	6	287303	286521	287 kb
		13258268	13259050			SOVchr6_13545584	13545584		287316	286534	287 kb
		13258268	13259050			SOVchr6_13545609	13545609		287341	286559	287 kb
		13258268	13259050			SOVchr6_13545882	13545882		287614	286832	287 kb
		13258268	13259050			SOVchr6_13545887	13545887		287619	286837	287 kb
*SOV6g004530*	6	13272920	13276376	3457	Putative receptor- like protein kinase	SOVchr6_13545571	13545571	6	272651	269195	269 kb
		13272920	13276376			SOVchr6_13545584	13545584		272664	269208	269 kb
		13272920	13276376			SOVchr6_13545609	13545609		272689	269233	269 kb
		13272920	13276376			SOVchr6_13545882	13545882		272962	269506	270 kb
		13272920	13276376			SOVchr6_13545887	13545887		272967	269511	270 kb
*SOV6g004550*	6	13277985	13281376	3392	putative receptor- like protein kinase At4g00960	SOVchr6_13545571	13545571	6	267586	264195	264 kb
		13277985	13281376			SOVchr6_13545584	13545584		267599	264208	264 kb
		13277985	13281376			SOVchr6_13545609	13545609		267624	264233	264 kb
		13277985	13281376			SOVchr6_13545882	13545882		267897	264506	265 kb
		13277985	13281376			SOVchr6_13545887	13545887		267902	264511	265 kb
*SOV6g004560*	6	13339413	13344361	4949	Pentatricopeptide repeat	SOVchr6_13545571	13545571	6	206158	201210	201 kb
		13339413	13344361			SOVchr6_13545584	13545584		206171	201223	201 kb
		13339413	13344361			SOVchr6_13545609	13545609		206196	201248	201 kb
		13339413	13344361			SOVchr6_13545882	13545882		206469	201521	202 kb
		13339413	13344361			SOVchr6_13545887	13545887		206474	201526	202 kb

On chromosome 6, five associated SNPs clustered within a narrow genomic region (~13.545–13.546 Mb), forming distinct LD blocks as identified by Haploview analysis (**Supplementary Figure 5**). Four annotated candidate genes were prioritized within the extended associated interval on chromosome 6, including *SOV6g004520* (cysteine-rich receptor-like kinase), *SOV6g004530* and *SOV6g004550* (putative receptor-like protein kinases), and *SOV6g004560* (pentatricopeptide repeat protein).

The remaining genes identified within LD regions were either unannotated or lacked clear functional relevance to bolting and were not prioritized further.

### 3.5 GP using random SNP subsets

**Figure 4:**
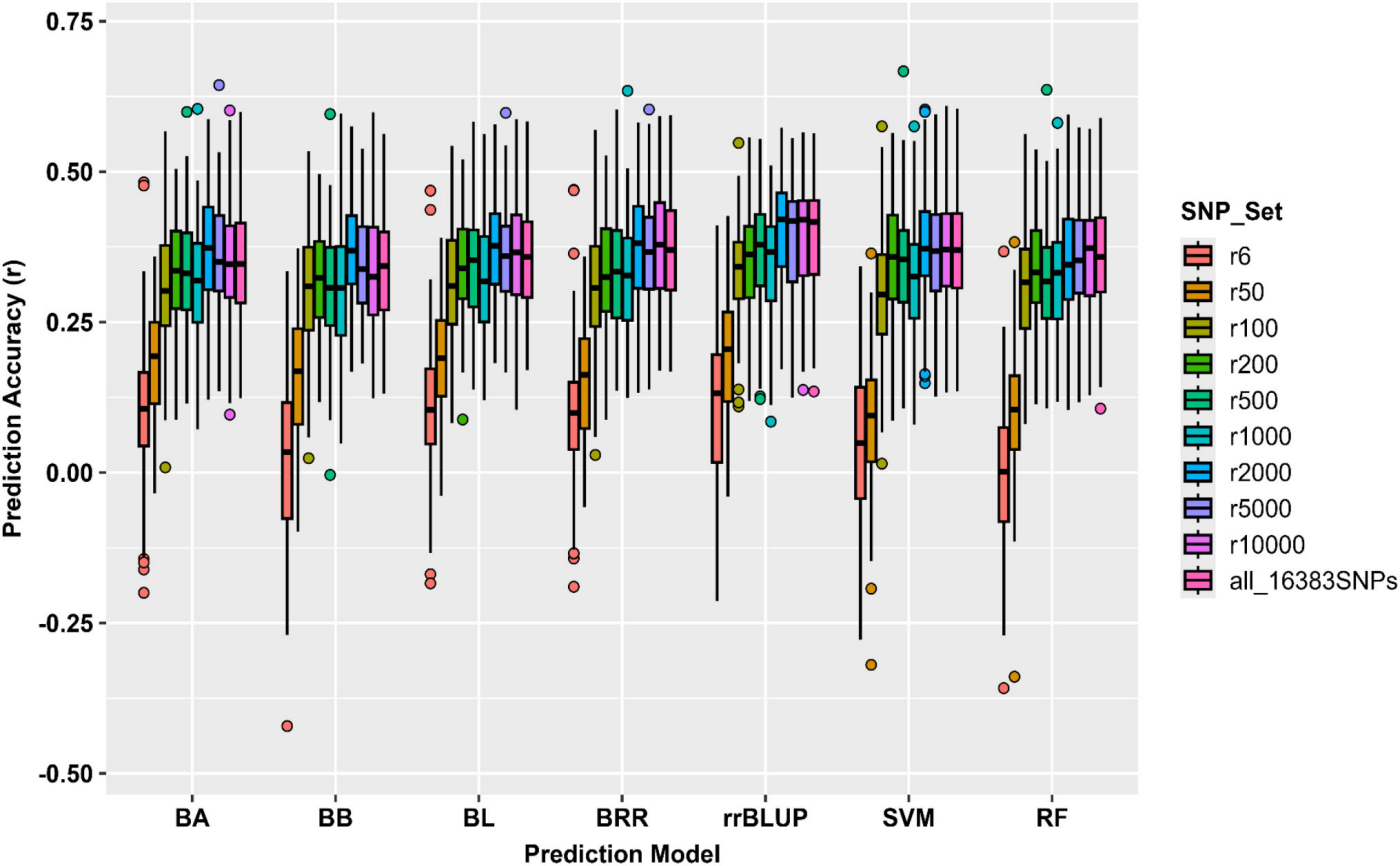
GP accuracy (*r*-value) of the bolting trait in 295 spinach accessions conducted in GAPIT3 using 16,563 SNP markers. Seven prediction models (BA, BB, BL, BRR, rrBLUP, SVM, and RF) were evaluated across ten SNP subsets (r6 - r10000 and all_ 16563 SNPs). Each boxplot represents the distribution of prediction accuracies (*r*) for the respective model and SNP set.

Prediction accuracy increased progressively with SNPs density across all models (**Supplementary Table 3****;** **Figure 4**). The mean correlation coefficient (*r*) rose from 0.07 for r6 to 0.37 for r2000. Beyond this density (r5000-all_16,563), accuracy stabilized at *r* ≈ 0.36, suggesting that additional markers contributed minimal improvement. Among models, rrBLUP and BRR achieved the highest accuracies (*r* ≈ 0.39), whereas BA, BB, and BL produced similar values (*r* ≈ 0.33 – 0.37). The non- parametric models RF and SVM performed comparably (*r* ≈ 0.35 – 0.37), indicating convergence between linear and non-linear algorithms. SEs were consistently low (*SE* ≈ 0.09 – 0.10), confirming stable performance (**Supplementary Figure 7**). These results suggest that approximately 2,000 randomly selected SNPs capture most of the additive genetic variance contributing to bolting prediction in spinach.

### 3.6 GP using GWAS-derived SNP sets

**Figure 5:**
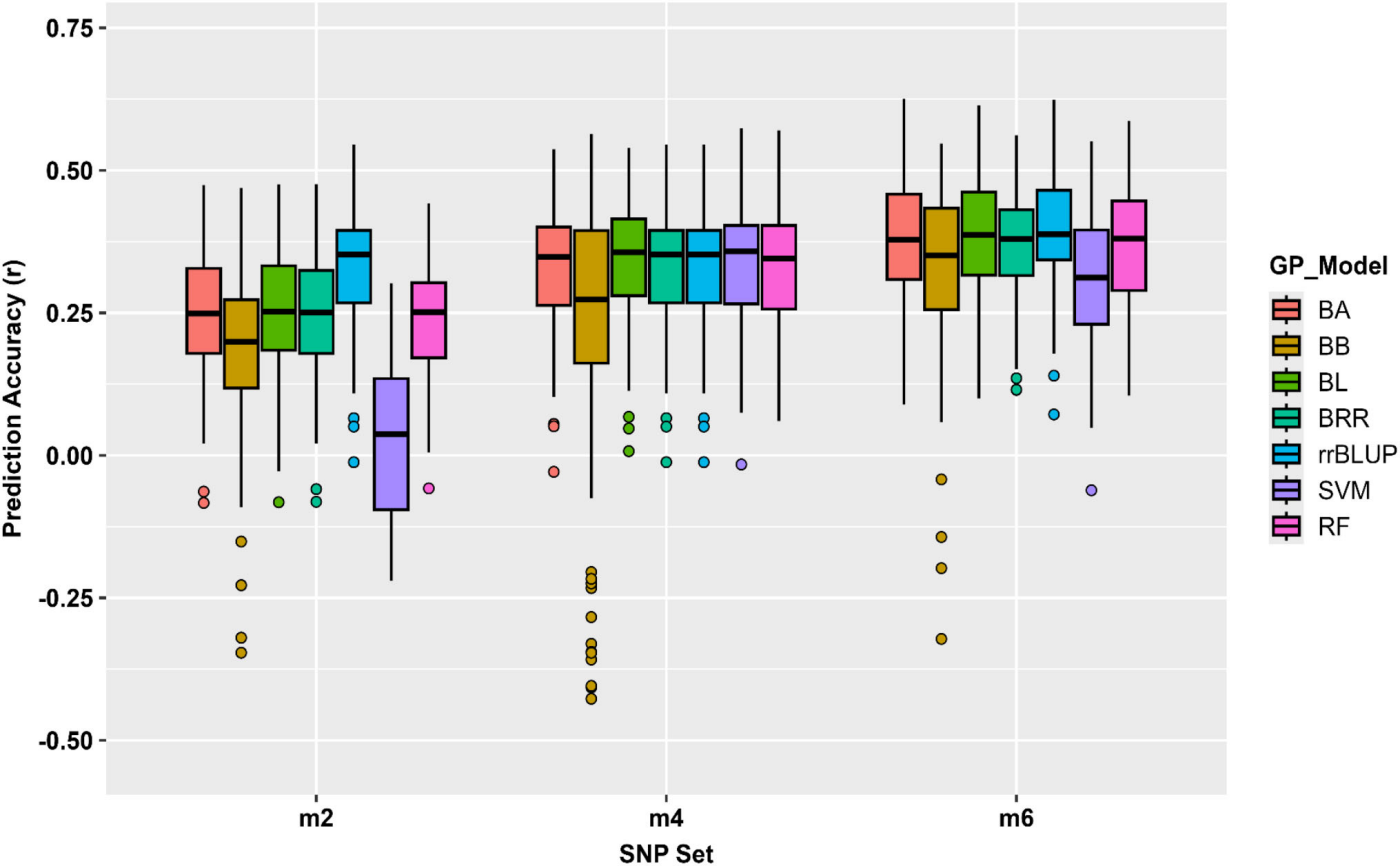
GP accuracy (*r*-value) for bolting trait using four GWAS-derived SNP sets (m2: 2 markers; m4: 4 markers; m6: 6 markers). Prediction was conducted through cross-population analysis using five-fold cross-validation (training: validation = 4:1) across seven GP models: BA, BB, BL, BRR, rrBLUP, RF, and SVM. Each boxplot represents the distribution of prediction accuracy for the respective model and SNP set.

Prediction accuracy varied across GWAS-derived marker sets and genomic prediction models (**Supplementary Table 4****;** **Figure 5**). Notably, mean *r*-values increased with the number of associated SNPs, with the m6 marker set showing the highest overall performance (mean *r*-value = 0.36), followed by m4 (mean *r*-value = 0.32) and m2 (mean *r*-value = 0.22). This pattern was consistent across most genomic prediction models.

Across models, rrBLUP achieved the highest average prediction accuracy (mean *r*-value = 0.35), followed by BL and BRR (mean *r*-value ≈ 0.32 – 0.31). In contrast, BA, BB, RF, and SVM exhibited comparatively lower but consistent performance (mean *r*-value ≈ 0.22 – 0.32). The highest single- model accuracy was observed for rrBLUP using the m6 marker set (*r*-value = 0.39).

Prediction uncertainty was uniformly low across marker sets and models. Standard errors ranged from 0.007 to 0.017, with mean SE values below 0.01 for all models, indicating stable and reproducible prediction performance. These results indicate that increasing the number of GWAS- derived SNPs from two to six improved genomic prediction accuracy, whereas differences among prediction models were comparatively modest.

### 3.7 GP using GWAS-derived SNPs from the training population (80%)

**Figure 6:**
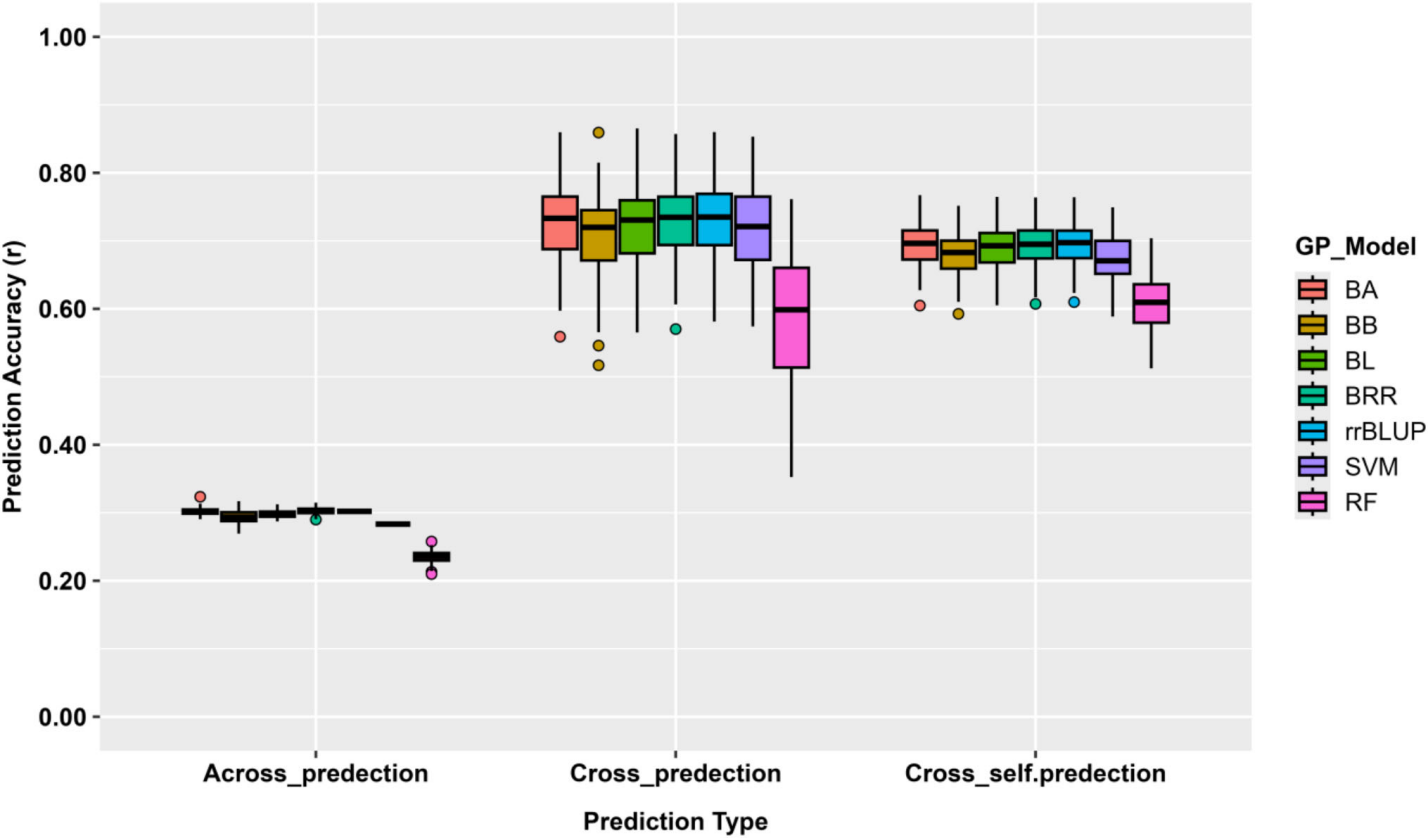
GP accuracy (*r*-value) for bolting trait using GWAS-derived SNP markers. Three prediction strategies were evaluated: Across-prediction (80% training set, 236 accessions, used to predict the remaining 20%, 59 accessions), Cross-prediction (SNPs from all training sets combined to predict the full panel of 295 accessions), and Cross_self-prediction (training sets used to predict themselves). Seven GP models (BA, BB, BL, BRR, rrBLUP, SVM, and RF) were evaluated. Each boxplot represents the distribution of prediction accuracy for the respective model and SNP set.

Prediction accuracies varied across the three evaluation scenarios (**Supplementary Table 4****;** **Figure 6**). In the across-population prediction (5-fold validation), the mean correlation coefficient was low (*r*-value ≈ 0.29), reflecting reduced transferability between training and validation sets. Accuracy increased markedly in the across-self prediction, where 80% of accessions were used for training and 20% for validation, reaching an average *r*-value = 0.69 (range: 0.57 – 0.79). Cross-population prediction achieved comparable accuracy (*r*-value = 0.70). The BRR and rrBLUP models performed best (*r*-value = 0.73). SEs were low across all models (SE ≈ 0.04 – 0.11), confirming stable predictions. Prediction accuracy was strongly influenced by population relatedness, being higher when training and validation sets were genetically similar and lower when they were genetically distinct.

### 3.8 GP using GAGBLUP in GAPIT3 with GWAS-derived SNP markers

**Figure 7:**
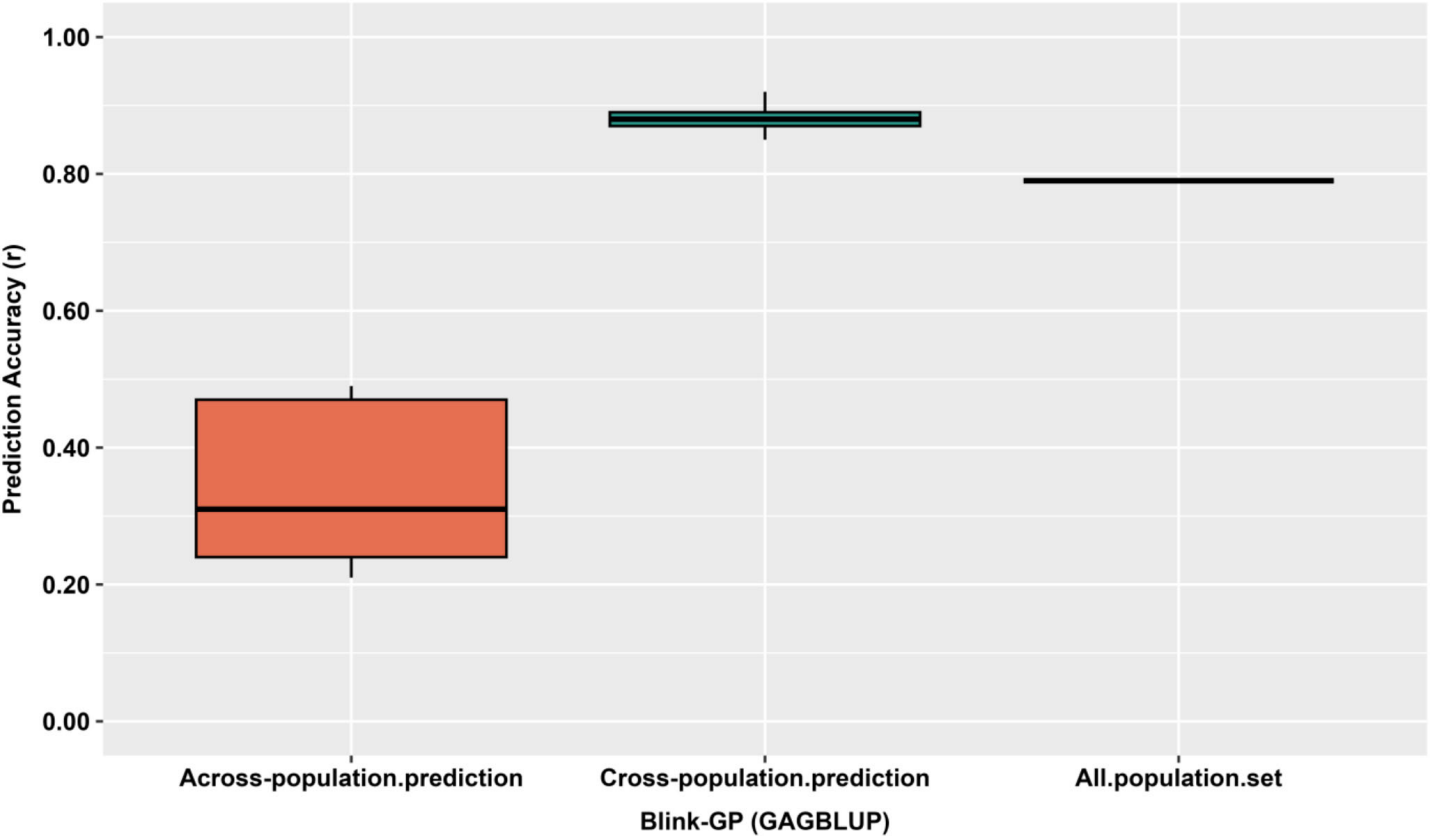
GP accuracy (*r*-value) for the bolting trait in 295 spinach accessions using the GAGBLUP (BLINK) model implemented in GAPIT3. Three prediction strategies were evaluated: Across-population prediction, in which GWAS-derived SNP markers from the training population (TP, 80%; 236 accessions) were used to predict the validation population (VP, 20%; 59 accessions); Cross-population prediction, where SNP markers from the TP (80%; 236 accessions) were used to predict genomic estimated breeding values (GEBVs) within the same training set; and All-population set, in which GWAS-derived SNP markers identified by BLINK were used to predict the entire population (295 accessions).

Genomic prediction using GWAS-derived SNP markers with the GAGBLUP model in GAPIT3 showed clear differences among prediction schemes (**Figure 7**). Prediction accuracy was highest for cross-population prediction, with a mean *r*-value of 0.88, followed by self-prediction using the full population (mean *r*-value = 0.79). In contrast, across-population prediction resulted in substantially lower accuracy (mean *r*-value = 0.34). These patterns indicate that GAGBLUP-based prediction using significant GWAS SNPs performs well when training and prediction populations overlap, whereas predictive ability declines when models are transferred across populations.

## 4 Discussion

### 4.1 Phenotypic variation in bolting trait

Bolting trait among the 295 spinach (*Spinacia oleracea* L.) accessions showed a bimodal distribution, indicating a discontinuous physiological response to floral induction rather than a gradual variation (Chitwood et al., 2016). This pattern suggests that, under controlled greenhouse conditions, the transition from vegetative to reproductive growth functions as a threshold trait primarily regulated by photoperiod sensitivity and temperature accumulation (Abolghasemi et al., 2021).

The predominance of early-bolting accessions from Southwest Asia (Afghanistan and Syria) and late- bolting accessions from temperate regions (Turkey, North Macedonia, and the United States) reflects regional adaptation to contrasting growing seasons and daylength regimes (Abolghasemi et al., 2021). Early bolting genotypes appear adapted to short spring seasons with rapidly increasing day length, whereas late-bolting accessions may carry alleles conferring delayed floral initiation under extended photo periods and moderate temperatures (Andrés and Coupland, 2012).

Comparable geographic differentiation in bolting behavior has been reported in other leafy crops such as lettuce (*Lactuca sativa*), sugar beet (*Beta vulgaris*), and Chinese cabbage (*Brassica rapa*), where vernalization and photoperiod cues jointly regulate floral transition (Andrés and Coupland, 2012; Pattison and Catalá, 2012; Song et al., 2015; Hong et al., 2021). In these species, allelic variation in genes controlling photoperiod and vernalization pathways defines distinct ecotypes adapted to local environments, for example winter and spring beet cultivars or temperate and subtropical lettuce types (Andrés and Coupland, 2012; Pin et al., 2012; Hong et al., 2021).

A similar adaptive pattern is plausible in spinach, where alleles delaying flowering enhance vegetative yield under long days, whereas early-flowering alleles facilitate seed production before exposure to high-temperature stress (Abolghasemi et al., 2021; Meng et al., 2022). The absence of an intermediate bolting group in the present study likely reflects the threshold nature of floral induction, in which bolting is triggered once a critical combination of photoperiod and temperature cues exceeds a physiological limit (Chitwood et al., 2016). Under uniform greenhouse conditions, environmental variation was minimized, thereby accentuating this binary response between early and late genotypes.

Similar bimodal patterns have been described in controlled photoperiod experiments in spinach and other long-day crops, supporting the hypothesis that bolting is governed by discrete genetic switches rather than continuous quantitative variation (Pin et al., 2012; Meng et al., 2022). The observed bimodality therefore most likely reflects underlying genetic differentiation between photoperiod- sensitive and photoperiod-insensitive alleles segregating within the population (Andrés and Coupland, 2012; Abolghasemi et al., 2021).

The moderate genomic heritability estimated for bolting time (33.13%), together with the higher residual variance (10.50%) relative to the genetic variance (5.20%), indicates that environmental factors contribute substantially to phenotypic variation in Spinacia oleracea. This variance partitioning suggests that although the transition to bolting is genetically regulated, the precise timing of floral induction remains strongly influenced by environmental cues such as photoperiod and temperature. Similar genotype – environment interactions regulating bolting have been reported in spinach and other long-day crops where flowering responses depend on both genetic sensitivity and environmental signals (Abolghasemi et al., 2021; Meng et al., 2022).

### 4.2 Population structure and genetic diversity

Analysis of genome-wide SNP variation revealed clear genetic stratification within the spinach panel, with the 295 accessions separating into three major genetic clusters (Q1–Q3). This pattern is consistent with previous studies reporting moderate subpopulation differentiation in global spinach germplasm (Abolghasemi et al., 2021; Meng et al., 2022; Alatawi et al., 2025).

The first three principal components accounted for 19.9% of the total genetic variance, a proportion comparable to that reported in earlier spinach studies and in other leafy vegetable crops with long histories of domestication and breeding selection (Pin et al., 2012; Chitwood et al., 2016; Ribera et al., 2020). Although individual components explained a relatively modest fraction of variance, the distinct separation among clusters indicates that a limited number of major axes capture the dominant genetic structure present in the panel. Similar patterns are commonly observed in cultivated germplasm collections shaped by regional differentiation accompanied by historical gene flow (Pin et al., 2012; Ribera et al., 2020).

Geographically, Q1 included Central and Western Asian accessions (Turkey, Afghanistan, and Iran), Q2 comprised South and East Asian materials, and Q3 represented European and North American accessions. This pattern aligns with the proposed domestication and dispersal history of spinach, which originated in ancient Persia and expanded eastward and westward along historical trade routes before secondary diversification in Europe (Rubatzky and Yamaguchi, 1997; Abolghasemi et al., 2021). The intermediate position of several Indian and Chinese accessions between Q1 and Q2 suggests gene flow during eastward expansion, consistent with findings from recent resequencing effort (Hirakawa et al., 2021).

This structured yet interconnected genetic background reflects the combined effects of historical migration and breeding selection on spinach diversity (Abolghasemi et al., 2021; Hirakawa et al., 2021). Controlling for this population stratification is essential for reliable interpretation of GWAS results and detection of alleles linked to adaptive traits.

### 4.3 GWAS of bolting trait

GWAS identified seven loci significantly associated with bolting trait, distributed across chromosomes 2, 4, and 6 (**Table 1**). The concordance among the five statistical models (GLM, MLM, MLMM, FarmCPU, and BLINK) indicated the robustness of these associations and confirmed adequate correction for population structure and kinship (Liu et al., 2016; Huang et al., 2019; Wang and Zhang, 2021). Combining single-locus (GLM, MLM) and multi-locus (MLMM, FarmCPU, BLINK) approaches allowed the detection of both major- and moderate-effect loci, a strategy previously shown to enhance resolution for complex traits such as flowering time (Segura et al., 2012; Huang et al., 2019).

A prominent association peak was detected on chromosome 6, where five adjacent SNPs (SOVchr6_13545571-SOVchr6_13545887) exceeded the genome-wide threshold and collectively explained the largest PVE (2 - 21%). These SNPs showed clear allelic differentiation between early- and late-bolting accessions, suggesting a genomic region in strong LD that may contain causal variants controlling floral transition (Chitwood et al., 2016; Abolghasemi et al., 2021). Earlier spinach GWAS studies also identified major flowering- and bolting-related loci on chromosome 6, indicating that this region represents a conserved hotspot for photoperiod sensitivity and reproductive development.

In addition to chromosome 6, significant associations were identified on chromosomes 2 and 4. The locus on chromosome 2 (SOVchr2_3254529; −log_10_(*P)* = 5.83; PVE = 10.93%) was supported by multiple models and overlaps previously reported regions associated with morphological and developmental traits, including leaf architecture and flowering behavior (Rasheed et al., 2017; Abolghasemi et al., 2021; Meng et al., 2022). Regions on this chromosome also regulate photoperiod and temperature responses in other long-day crops such as sugar beet and lettuce, which share conserved flowering pathways (Pin et al., 2012; Song et al., 2015). The weaker signal detected on chromosome 4 (SOVchr4_30429857) under the FarmCPU model may represent a minor or condition-dependent locus. Notably, chromosome 4 contains the spinach sex-determining region and genes involved in reproductive organ development, which could explain its occasional co- localization with bolting-related variation (Hong et al., 2021; Zhao et al., 2021).

The proportion of PVE by individual SNPs (2-21%) aligns with the polygenic and moderately heritable nature of flowering-time traits in leafy crops (Hong et al., 2021; Meng et al., 2022). Similar genetic architectures, where a few key regulators interact with multiple small-effect modifiers, have been described in lettuce, beet, and Chinese cabbage (Pin et al., 2012; Song et al., 2015; Hong et al., 2021). The close alignment between observed and expected *P*-values in QQ plots further supports the reliability of these associations and the stability of the models (Huang et al., 2019; Hong et al., 2021).

The results indicate that chromosome 6 harbors the major loci governing bolting in spinach, while chromosomes 2 and 4 contribute minor associations. This multi-model GWAS framework provides a solid foundation for refining association signals and identifying candidate genes underlying flowering control (Abolghasemi et al., 2021; Meng et al., 2022).

### 4.4 Candidate gene identification

Fifteen genes were identified within LD blocks surrounding seven SNPs significantly associated with bolting trait. Functional annotation and comparative analysis prioritized five biologically plausible candidates, four of which cluster within a high-LD region on chromosome 6 (132–135 Mb). This clustering suggests a potential genomic hotspot influencing floral transition in *Spinacia oleracea*. The candidate loci encode proteins involved in RNA processing, signal transduction, and organellar communication, functional categories repeatedly implicated in flowering-time regulation across plant species. RNA-mediated regulation emerges as a plausible mechanism contributing to bolting variation. *SOV4g014340*, encoding a glycine-rich RNA-binding protein (GRP), represents a strong candidate given the established roles of *GRPs* in post-transcriptional and circadian regulation of flowering pathways. In *Arabidopsis thaliana*, glycine-rich RNA-binding proteins such as *AtGRP7* have been shown to influence floral transition through post-transcriptional regulation of flowering pathways, including effects on *FLC* expression and circadian timing (Streitner et al., 2008). In addition, overexpression of the related *GRP, GRDP2*, has been reported to accelerate bolting in lettuce, supporting a conserved role for *GRPs* in regulating the vegetative-to-reproductive transition across species (Hong et al., 2021). Consistent with these findings, spinach transcriptomic studies report co- expression of GRP-like transcripts with *COL* and *FT* homologs during bolting onset (Wu et al., 2024; Shahbaz et al., 2025), supporting a conserved RNA-level regulatory contribution to the vegetative-to- reproductive transition.

Signaling-related genes also represent credible candidates for bolting regulation. *SOV6g004520*, *SOV6g004530*, and *SOV6g004550* encode cysteine-rich receptor-like kinases (CRKs), which integrate environmental, redox, and hormonal cues. In *Arabidopsis*, CRK family members participate in stress-responsive developmental regulation, and disruption of *CRK2* delays flowering and alters gibberellin sensitivity (Bourdais et al., 2015). Similar enrichment of receptor-like kinases near flowering-time quantitative trait loci has been reported in sugar beet, indicating conservation of this signaling architecture within Amaranthaceae crops (Hirakawa et al., 2021). The co-localization of multiple CRKs within the chromosome 6 LD block suggests a regulatory module linking environmental perception to floral induction.

A further candidate, *SOV6g004560*, encodes a pentatricopeptide repeat (PPR) protein, implicating organellar to nuclear communication in bolting regulation. Although PPR proteins primarily function in organellar RNA processing, retrograde signaling from chloroplasts and mitochondria has been shown to influence flowering time. In *Arabidopsis*, mutation of the mitochondrial PPR gene *PRECOCIOUS1* accelerates flowering through repression of *FLC*, potentially involving changes in abscisic acid–related signaling pathways associated with retrograde communication (Leister, 2012; Emami and Kempken, 2019). By analogy, *SOV6g004560* may modulate developmental timing by coordinating organellar energy status with nuclear gene expression.

In contrast, several genes within the associated LD intervals encode proteins with fundamental cellular roles, including amino acid metabolism, nucleotide biosynthesis, and DNA mismatch repair, such as *SOV2g000950*, *SOV4g014330*, and *SOV6g004570*. These functions have not been linked to flowering-time regulation in plant systems, and their association with bolting signals is most parsimoniously explained by physical linkage rather than causal involvement (Dion et al., 2007; Mao et al., 2021).

The current findings suggest that bolting variation in spinach is shaped by regulatory networks integrating RNA processing, environmental signaling, and organellar communication, rather than solely by canonical flowering-time genes. Although the prioritized candidate genes are biologically plausible based on genomic evidence and established roles in related species, systematic expression profiling across developmental stages is required to fully elucidate specific regulatory mechanisms and confirm differential activity during the floral transition. Consequently, this study establishes a robust framework for future functional validation, including eQTL mapping and targeted genetic analyses, to clarify the molecular basis of bolting control in *Spinacia oleracea.*

### 4.5 GP of bolting trait

GP analyses revealed consistent trends showing how marker density, biological informativeness, and population relatedness influence predictive ability in spinach. Prediction accuracy increased with marker number up to approximately 2,000 SNPs and then plateaued at *r* ≈ 0.36, indicating that a moderate number of loci captured most of the additive variance for bolting trait. While accuracy stabilized at this threshold, all final prediction models were implemented using the full set of 16,563 SNPs to ensure comprehensive genomic coverage and robust kinship estimation. Similar saturation patterns have been reported in maize, ryegrass, and sugar beet, where accuracy rises sharply with increasing marker density before stabilizing once genome-wide relationships are adequately represented (Zhang et al., 2017; Hong et al., 2021). The stability of accuracies beyond this threshold suggests moderate LD in spinach, sufficient for reliable kinship estimation without excessive marker redundancy.

Among models, rrBLUP and BRR achieved the highest accuracies (*r* ≈ 0.39), which is consistent with the infinitesimal model assumption where marker effects are treated as random variables following a Gaussian distribution with equal variance (Endelman, 2011). Although BA, BB, and BL, and machine learning approaches (RF and SVM) produced similar results, they did not outperform the linear additive models. These findings confirm that additive genetic variance is the primary driver of bolting regulation in the spinach population studied. Furthermore, since non-linear models like RF and SVM, which are theoretically better at capturing complex epistasis, did not provide a significant advantage, it suggests that non-additive contributions are relatively minor for the bolting trait in the current genetic background (Azodi et al., 2019). This observation is consistent with previous findings where linear models often match or outperform machine learning approaches in populations with moderate size and high LD (Heffner et al., 2009; Crossa et al., 2017).

Prediction using GWAS-derived marker sets (m2, m4, m6) produced moderate but consistent accuracies (*r* ≈ 0.36 – 0.37) across models. Notably, the m6 subset achieved a peak accuracy of *r* = 0.39 using the rrBLUP model, a performance that perfectly aligns with our whole-genome analysis (discussed above), where accuracies also plateaued at *r* ≈ 0.39 once marker density reached 2,000 SNPs. The stability of the rrBLUP and BRR models aligns with their underlying design to capture additive effects within an infinitesimal architecture. These findings reinforce the conclusion that bolting in this population is primarily driven by additive genetic effects, which can be effectively captured by a small number of high- impact loci.

These results facilitate a two-part genomic breeding strategy (Gaynor et al., 2017), whereby the breeding program is bifurcated into a population improvement component and a product development component. Under this framework, the m6 marker set serves as a high-efficiency tool for initial population improvement, enabling cost-effective, large-scale screening to increase the frequency of favorable alleles and cull undesirable genotypes at an early stage. Subsequently, whole-genome models provide the high-precision prediction required for the product development phase, where final elite selections are performed to identify superior varieties for release. This tiered approach is supported by the accuracy plateau observed in rice (*Oryza sativa*), where as few as 7 GWAS-derived markers achieved a prediction accuracy (*r* = 0.63) nearly equivalent to the full 38,425-marker panel (*r* = 0.64), further justifying the use of limited, prioritized loci for large-scale germplasm management (Spindel et al., 2016). Ultimately, while m6 facilitates rapid, cost-effective cycles of population improvement, a strategy that can deliver up to 2.4 times more genetic gain per unit cost (Gaynor et al., 2017), the integration of full- genome information remains essential for the final optimization of genetic gain in *Spinacia oleracea*.

When GWAS-derived SNPs from the training population (80%) were used, prediction accuracy varied across validation frameworks, reflecting the influence of population relatedness. Across- population prediction resulted in lower accuracy (*r* ≈ 0.29), indicating limited transferability of marker effects between genetically distinct groups, whereas within-population validation of the same 80/20 splits achieved the highest accuracy (*r* ≈ 0.69), consistent with shared allele frequencies and LD patterns. The combined cross-population framework also maintained high accuracy (*r* ≈ 0.70), likely because pooling GWAS signals across replicates captured stable and reproducible loci (Crossa et al., 2014; Spindel et al., 2016). The superior performance of rrBLUP and BRR (*r* ≈ 0.73) reinforces that bolting trait is governed primarily by additive effects (Heffner et al., 2009; Crossa et al., 2017).

The findings indicate that marker informativeness and population structure jointly shape genomic predictability, emphasizing the need for genetically representative training populations to improve model transferability and support reliable GP-based selection in spinach breeding.

Within this broader genomic prediction context, evaluating GWAS-assisted prediction through a GAGBLUP framework offers additional insight into how biologically associated loci contribute to bolting trait predictability under alternative evaluation designs. The clear stratification in predictive performance across scenarios indicates that, even when derived from a single germplasm panel, accuracy is shaped primarily by training representation and validation design rather than true genetic divergence among accessions. Higher accuracy observed under internally consistent evaluation reflects closer alignment between loci identified through GWAS and the phenotypic variance captured during GEBV estimation, whereas the decline under stricter validation highlights the sensitivity of GWAS-assisted prediction to reduced training size and reliance on a limited set of associated markers (Selga et al., 2022; Xu et al., 2024; Ma et al., 2025). These patterns are consistent with the quantitative and predominantly additive genetic architecture of bolting, where multiple loci of small to moderate effect collectively influence developmental timing, limiting the transferability of marker effects when sampling representation is constrained (Heffner et al., 2009; Crossa et al., 2017). These results suggest that GWAS-assisted genomic prediction using GAGBLUP is most informative for within-panel interpretation and early screening against premature bolting, while its performance across more stringent validation frameworks remains contingent on training representativeness and marker coverage. In summary, this study not only clarifies the genomic basis of bolting trait in spinach using GWAS-assisted genomic prediction, but also identifies informative marker sets that support reliable prediction under appropriate evaluation frameworks.

## 5 Conclusion

GWAS analysis was integrated with genomic prediction to investigate the genetic architecture of bolting trait in spinach (*Spinacia oleracea* L.) using a panel of 295 accessions with 16,563 high- quality SNPs. Seven significant loci were detected across chromosomes 2, 4, and 6, with a prominent association region on chromosome 6. Within the linkage disequilibrium intervals surrounding these loci, several candidate genes were identified, including *SOV6g004520*, *SOV6g004530*, *SOV6g004550*, and *SOV6g004560*, indicating that this region represents an important genomic hotspot associated with *bolting* variation.

GP analyses indicated that bolting trait is predominantly influenced by additive genetic effects, with prediction accuracy increasing with marker density up to approximately 2,000 SNPs before reaching a plateau. GWAS-derived SNP marker sets supported stable prediction under training-consistent evaluation designs, whereas reduced accuracy under across-population validation emphasized the importance of representative training populations for reliable model transferability. The current findings clarify the genetic basis of bolting trait in spinach and provide validated loci and marker sets that are directly applicable to marker-assisted and genomic selection for improving bolting management in breeding programs.

## Data Availability

The whole genome resequencing (WGR) data aligned to the reference genome is publicly available at NCBI Sequence Read Archive (SRA) under BioProject ID PRJNA860974 (https://www.ncbi.nlm.nih.gov/sra/?term=PRJNA860974). The SNP data generated in this study are available at the Figshare repository (https://doi.org/10.6084/m9.figshare.30983827). Relevant SNP results are presented within the article and its **Supplementary Material**.
